# (*E*)-*N*′-[1-(4-Chloro­phen­yl)ethyl­idene]-2-hydroxy­benzohydrazide

**DOI:** 10.1107/S1600536809002311

**Published:** 2009-01-23

**Authors:** Man-Lin Li, Xianqiang Huang, Ruo-Kun Feng

**Affiliations:** a College of Science, Northwest A and F University, Yangling, Shaanxi 712100, People’s Republic of China; b College of Chemistry and Chemical Engineering, Liaocheng University, Shandong 252059, People’s Republic of China

## Abstract

In the title compound, C_15_H_13_ClN_2_O_2_, the dihedral angle between the two benzene rings is 7.0 (1)°. An intra­molecular N—H⋯O hydrogen bond is present and inter­molecular O—H⋯O hydrogen bonds link the mol­ecules into chains along [001].

## Related literature

For related literature, see: Sumita *et al.* (1999 ▶). For the crystal structure of the closely related compound (*E*)-2-hydr­oxy-*N*′-(2-naphthyl­methyl­ene)benzohydrazide, see: Qiu *et al.* (2006 ▶).
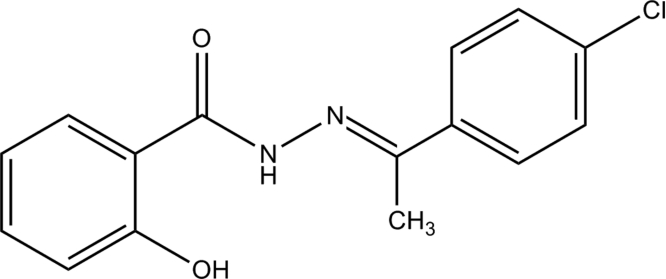


## Experimental

### 

#### Crystal data

C_15_H_13_ClN_2_O_2_
*M* *_r_* = 288.72
Monoclinic, 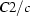

*a* = 27.900 (3) Å
*b* = 7.880 (1) Å
*c* = 13.4899 (15) Å
β = 113.530 (2)°
*V* = 2719.2 (5) Å^3^
*Z* = 8
Mo *K*α radiation
μ = 0.28 mm^−1^
*T* = 293 (2) K
0.35 × 0.17 × 0.07 mm

#### Data collection

Bruker SMART CCD diffractometer
Absorption correction: multi-scan (*SADABS*; Sheldrick, 1996 ▶) *T*_min_ = 0.907, *T*_max_ = 0.980
4744 measured reflections
1663 independent reflections
901 reflections with *I* > 2σ(*I*)
*R*_int_ = 0.082
θ_max_ = 22.0°

#### Refinement

*R*[*F* ^2^ > 2σ(*F* ^2^)] = 0.065
*wR*(*F* ^2^) = 0.266
*S* = 0.96
1663 reflections
182 parameters
H-atom parameters constrained
Δρ_max_ = 0.33 e Å^−3^
Δρ_min_ = −0.37 e Å^−3^

### 

Data collection: *SMART* (Bruker, 1997 ▶); cell refinement: *SAINT* (Bruker, 1997 ▶); data reduction: *SAINT*; program(s) used to solve structure: *SHELXS97* (Sheldrick, 2008 ▶); program(s) used to refine structure: *SHELXL97* (Sheldrick, 2008 ▶); molecular graphics: *SHELXTL* (Sheldrick, 2008 ▶); software used to prepare material for publication: *SHELXTL*.

## Supplementary Material

Crystal structure: contains datablocks I, global. DOI: 10.1107/S1600536809002311/bi2333sup1.cif

Structure factors: contains datablocks I. DOI: 10.1107/S1600536809002311/bi2333Isup2.hkl

Additional supplementary materials:  crystallographic information; 3D view; checkCIF report

## Figures and Tables

**Table 1:** Hydrogen-bond geometry (Å, °)

*D*—H⋯*A*	*D*—H	H⋯*A*	*D*⋯*A*	*D*—H⋯*A*
N1—H1⋯O2	0.86	1.96	2.645 (6)	135
O2—H2⋯O1^i^	0.82	1.92	2.676 (6)	153

Symmetry code: (i) 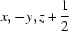.

**Fig. 1.**
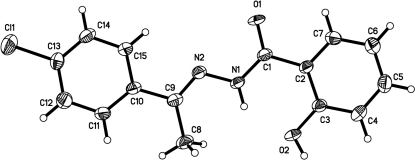
Molecular structure with displacement ellipsoids drawn at 30% probability for non-H atoms.

## supplementary crystallographic information

### Comment

Salicyloyl hydrazide is an important organic intermediate, which can act as moulding board in inorganic complexes (Sumita *et al.*, 1999). The title compound was obtained by reaction of salicyloyl hydrazide and 1-(4-chlorophenyl)ethanone. The bond lengths and angles are normal and comparable to those in the previously reported compound (*E*)-2-hydroxy-*N*'-(2-naphthylmethylene)-benzohydrazide (Qiu *et al.*, 2006).

In the crystal structure, typical intramolecular N—H···O hydrogen bonds exist, and intermolecular O—H···O hydrogen bonds link the molecules into one-dimensional chains along [001].

### Experimental

Salicyloyl hydrazide (0.3 mmol) and freshly prepared 1-(4-chlorophenyl)ethanone (0.3 mmol) were mixed in a 50 ml flask. After stirring for 30 min at 353 K, the mixture was cooled slowly to room temperature and the product was recrystallized from ethanol, affording the title compound as a green crystalline solid. Elemental analysis calculated: C 62.40, H 4.54, N 9.70%; found: C 62.58, H 4.45, N 9.64%.

### Refinement

All H atoms were placed in geometrically idealized positions (N—H = 0.86, O—H = 0.82 and C—H = 0.93–0.96 Å) and refined as riding with *U*_iso_(H) = 1.2 *U*_eq_(C,O,N). Diffraction was relatively weak and the data are truncated to 0.95 Å resolution, with 901 of 1663 unique reflections (ca 54%) observed. As a consequence, the refined structure is of relatively low precision.

### Crystal data

**Table:**

C_15_H_13_ClN_2_O_2_	*F*(000) = 1200
*M**_r_* = 288.72	*D*_x_ = 1.411 Mg m^−^^3^
Monoclinic, *C*2/*c*	Mo *K*α radiation, λ = 0.71073 Å
Hall symbol: -C 2yc	Cell parameters from 951 reflections
*a* = 27.900 (3) Å	θ = 2.7–25.1°
*b* = 7.880 (1) Å	µ = 0.28 mm^−^^1^
*c* = 13.4899 (15) Å	*T* = 293 K
β = 113.530 (2)°	Block, green
*V* = 2719.2 (5) Å^3^	0.35 × 0.17 × 0.07 mm
*Z* = 8	

### Data collection

**Table:**

Bruker SMART CCD diffractometer	1663 independent reflections
Radiation source: fine-focus sealed tube	901 reflections with *I* > 2σ(*I*)
graphite	*R*_int_ = 0.082
φ and ω scans	θ_max_ = 22.0°, θ_min_ = 1.6°
Absorption correction: multi-scan (*SADABS*; Sheldrick, 1996)	*h* = −29→20
*T*_min_ = 0.907, *T*_max_ = 0.980	*k* = −8→8
4744 measured reflections	*l* = −14→14

### Refinement

**Table:**

Refinement on *F*^2^	Primary atom site location: structure-invariant direct methods
Least-squares matrix: full	Secondary atom site location: difference Fourier map
*R*[*F*^2^ > 2σ(*F*^2^)] = 0.065	Hydrogen site location: inferred from neighbouring sites
*wR*(*F*^2^) = 0.266	H-atom parameters constrained
*S* = 0.96	*w* = 1/[σ^2^(*F*_o_^2^) + (0.1726*P*)^2^] where *P* = (*F*_o_^2^ + 2*F*_c_^2^)/3
1663 reflections	(Δ/σ)_max_ < 0.001
182 parameters	Δρ_max_ = 0.33 e Å^−^^3^
0 restraints	Δρ_min_ = −0.37 e Å^−^^3^

### Special details

**Table:**

Geometry. All e.s.d.'s (except the e.s.d. in the dihedral angle between two l.s. planes) are estimated using the full covariance matrix. The cell e.s.d.'s are taken into account individually in the estimation of e.s.d.'s in distances, angles and torsion angles; correlations between e.s.d.'s in cell parameters are only used when they are defined by crystal symmetry. An approximate (isotropic) treatment of cell e.s.d.'s is used for estimating e.s.d.'s involving l.s. planes.
Refinement. Refinement of *F*^2^ against ALL reflections. The weighted *R*-factor *wR* and goodness of fit *S* are based on *F*^2^, conventional *R*-factors *R* are based on *F*, with *F* set to zero for negative *F*^2^. The threshold expression of *F*^2^ > σ(*F*^2^) is used only for calculating *R*-factors(gt) *etc*. and is not relevant to the choice of reflections for refinement. *R*-factors based on *F*^2^ are statistically about twice as large as those based on *F*, and *R*- factors based on ALL data will be even larger.

### Fractional atomic coordinates and isotropic or equivalent isotropic displacement parameters (Å^2^)

**Table:**

	*x*	*y*	*z*	*U*_iso_*/*U*_eq_	
Cl1	−0.16974 (8)	0.5453 (3)	−0.40292 (16)	0.0745 (9)	
N1	0.07264 (19)	0.1148 (7)	0.0780 (4)	0.0390 (15)	
H1	0.0712	0.1193	0.1404	0.047*	
N2	0.0332 (2)	0.1842 (7)	−0.0112 (4)	0.0372 (15)	
O1	0.11636 (19)	0.0271 (8)	−0.0221 (4)	0.0714 (19)	
O2	0.11905 (18)	0.0618 (6)	0.2888 (3)	0.0531 (15)	
H2	0.1252	0.0612	0.3535	0.080*	
C1	0.1133 (2)	0.0401 (10)	0.0666 (5)	0.0422 (19)	
C2	0.1557 (2)	−0.0358 (9)	0.1654 (5)	0.0400 (18)	
C3	0.1585 (2)	−0.0201 (9)	0.2719 (5)	0.0405 (19)	
C4	0.2007 (3)	−0.0898 (10)	0.3581 (5)	0.050 (2)	
H4	0.2027	−0.0789	0.4283	0.060*	
C5	0.2391 (3)	−0.1741 (11)	0.3394 (6)	0.063 (2)	
H5	0.2671	−0.2198	0.3975	0.075*	
C6	0.2372 (3)	−0.1928 (11)	0.2365 (6)	0.060 (2)	
H6	0.2627	−0.2543	0.2242	0.071*	
C7	0.1960 (3)	−0.1174 (9)	0.1511 (6)	0.0457 (19)	
H7	0.1958	−0.1225	0.0820	0.055*	
C8	−0.0107 (3)	0.2598 (11)	0.1122 (5)	0.056 (2)	
H8A	−0.0043	0.1505	0.1465	0.084*	
H8B	−0.0452	0.2971	0.1005	0.084*	
H8C	0.0145	0.3397	0.1578	0.084*	
C9	−0.0057 (2)	0.2471 (9)	0.0044 (5)	0.0405 (18)	
C10	−0.0473 (3)	0.3259 (9)	−0.0938 (5)	0.0387 (18)	
C11	−0.0867 (3)	0.4294 (9)	−0.0915 (5)	0.046 (2)	
H11	−0.0883	0.4545	−0.0254	0.055*	
C12	−0.1242 (3)	0.4970 (10)	−0.1857 (6)	0.052 (2)	
H12	−0.1503	0.5674	−0.1824	0.063*	
C13	−0.1227 (3)	0.4592 (9)	−0.2842 (5)	0.0430 (19)	
C14	−0.0845 (3)	0.3575 (10)	−0.2901 (5)	0.052 (2)	
H14	−0.0827	0.3366	−0.3563	0.062*	
C15	−0.0479 (3)	0.2847 (9)	−0.1954 (5)	0.0450 (19)	
H15	−0.0236	0.2077	−0.2001	0.054*	

### Atomic displacement parameters (Å^2^)

**Table:**

	*U*^11^	*U*^22^	*U*^33^	*U*^12^	*U*^13^	*U*^23^
Cl1	0.0664 (15)	0.088 (2)	0.0516 (14)	0.0085 (12)	0.0054 (11)	0.0142 (12)
N1	0.039 (3)	0.058 (4)	0.023 (3)	0.000 (3)	0.016 (3)	0.002 (3)
N2	0.038 (3)	0.043 (4)	0.032 (3)	−0.001 (3)	0.016 (3)	0.001 (3)
O1	0.061 (3)	0.136 (6)	0.026 (3)	0.018 (3)	0.027 (3)	0.006 (3)
O2	0.062 (3)	0.078 (4)	0.025 (2)	0.006 (3)	0.023 (2)	0.005 (2)
C1	0.038 (4)	0.065 (5)	0.028 (4)	−0.011 (4)	0.018 (3)	−0.006 (3)
C2	0.036 (4)	0.060 (5)	0.028 (4)	−0.007 (4)	0.016 (3)	0.003 (3)
C3	0.038 (4)	0.053 (5)	0.036 (4)	−0.003 (3)	0.020 (3)	0.002 (3)
C4	0.057 (5)	0.053 (6)	0.034 (4)	−0.007 (4)	0.013 (4)	0.006 (4)
C5	0.045 (5)	0.084 (7)	0.054 (5)	0.009 (4)	0.014 (4)	0.015 (5)
C6	0.040 (5)	0.084 (7)	0.055 (5)	0.006 (4)	0.019 (4)	0.008 (5)
C7	0.051 (5)	0.046 (5)	0.047 (4)	−0.001 (4)	0.027 (4)	−0.004 (4)
C8	0.056 (5)	0.080 (7)	0.037 (4)	−0.001 (4)	0.025 (4)	0.006 (4)
C9	0.042 (4)	0.049 (5)	0.033 (4)	−0.013 (4)	0.018 (3)	−0.007 (3)
C10	0.042 (4)	0.050 (5)	0.028 (4)	−0.008 (4)	0.019 (3)	0.000 (3)
C11	0.044 (4)	0.059 (6)	0.038 (4)	0.001 (4)	0.020 (4)	−0.007 (4)
C12	0.048 (5)	0.057 (6)	0.052 (5)	0.003 (4)	0.021 (4)	0.006 (4)
C13	0.037 (4)	0.041 (5)	0.042 (4)	−0.005 (4)	0.006 (3)	0.006 (4)
C14	0.060 (5)	0.064 (6)	0.033 (4)	−0.003 (4)	0.021 (4)	0.002 (4)
C15	0.042 (4)	0.061 (6)	0.035 (4)	0.008 (4)	0.019 (3)	0.002 (4)

### Geometric parameters (Å, °)

**Table:**

Cl1—C13	1.752 (7)	C6—H6	0.930
N1—C1	1.340 (8)	C7—H7	0.930
N1—N2	1.379 (7)	C8—C9	1.518 (8)
N1—H1	0.860	C8—H8A	0.960
N2—C9	1.284 (8)	C8—H8B	0.960
O1—C1	1.237 (7)	C8—H8C	0.960
O2—C3	1.372 (8)	C9—C10	1.503 (9)
O2—H2	0.820	C10—C11	1.380 (9)
C1—C2	1.508 (9)	C10—C15	1.403 (8)
C2—C7	1.374 (9)	C11—C12	1.390 (9)
C2—C3	1.412 (9)	C11—H11	0.930
C3—C4	1.395 (9)	C12—C13	1.379 (10)
C4—C5	1.367 (10)	C12—H12	0.930
C4—H4	0.930	C13—C14	1.361 (10)
C5—C6	1.375 (10)	C14—C15	1.400 (9)
C5—H5	0.930	C14—H14	0.930
C6—C7	1.395 (9)	C15—H15	0.930
			
C1—N1—N2	119.4 (5)	C9—C8—H8B	109.5
C1—N1—H1	120.3	H8A—C8—H8B	109.5
N2—N1—H1	120.3	C9—C8—H8C	109.5
C9—N2—N1	116.2 (5)	H8A—C8—H8C	109.5
C3—O2—H2	109.5	H8B—C8—H8C	109.5
O1—C1—N1	122.4 (6)	N2—C9—C10	114.8 (5)
O1—C1—C2	119.3 (6)	N2—C9—C8	126.1 (6)
N1—C1—C2	118.3 (5)	C10—C9—C8	118.9 (6)
C7—C2—C3	117.8 (6)	C11—C10—C15	117.4 (6)
C7—C2—C1	117.3 (5)	C11—C10—C9	124.5 (5)
C3—C2—C1	124.8 (6)	C15—C10—C9	118.0 (6)
O2—C3—C4	120.8 (6)	C10—C11—C12	121.4 (6)
O2—C3—C2	119.2 (6)	C10—C11—H11	119.3
C4—C3—C2	120.0 (6)	C12—C11—H11	119.3
C5—C4—C3	120.0 (6)	C13—C12—C11	119.8 (7)
C5—C4—H4	120.0	C13—C12—H12	120.1
C3—C4—H4	120.0	C11—C12—H12	120.1
C4—C5—C6	121.3 (7)	C14—C13—C12	120.7 (6)
C4—C5—H5	119.3	C14—C13—Cl1	119.6 (5)
C6—C5—H5	119.3	C12—C13—Cl1	119.8 (6)
C5—C6—C7	118.4 (7)	C13—C14—C15	119.4 (6)
C5—C6—H6	120.8	C13—C14—H14	120.3
C7—C6—H6	120.8	C15—C14—H14	120.3
C2—C7—C6	122.3 (6)	C14—C15—C10	121.1 (7)
C2—C7—H7	118.8	C14—C15—H15	119.4
C6—C7—H7	118.8	C10—C15—H15	119.4
C9—C8—H8A	109.5		

### Hydrogen-bond geometry (Å, °)

**Table:**

*D*—H···*A*	*D*—H	H···*A*	*D*···*A*	*D*—H···*A*
N1—H1···O2	0.86	1.96	2.645 (6)	135
O2—H2···O1^i^	0.82	1.92	2.676 (6)	153

Symmetry codes: (i) *x*, −*y*, *z*+1/2.
